# AC Electrodeposition of PEDOT Films in Protic Ionic Liquids for Long-Term Stable Organic Electrochemical Transistors

**DOI:** 10.3390/molecules24224105

**Published:** 2019-11-14

**Authors:** Jianlong Ji, Xiaoxian Zhu, Dan Han, Mangmang Li, Qiang Zhang, Yang Shu, Zhengdong Cheng, Wendong Zhang, Er Hua, Shengbo Sang

**Affiliations:** 1 College of Information and Computer, Taiyuan University of Technology, Taiyuan 030024, China; zhuxiaoxian622@163.com (X.Z.); hd06520101@163.com (D.H.); 18634391812@163.com (M.L.); zhangqiang01@tyut.edu.cn (Q.Z.); wdzhang@tyut.edu.cn (W.Z.); 2 Department of Chemistry, Colleges of Sciences, Northeastern University, Shenyang 110819, China; shuyang@mail.neu.edu.cn; 3 Artie McFerrin Department of Chemical Engineering, Texas A&M University, College Station, TX 77843-3122, USA; zcheng@tamu.edu; 4 Chemical Science and Engineering College, North Minzu University, Ningxia 750021, China

**Keywords:** alternating current electrodeposition, organic electrochemical transistor, poly (3,4-ethylenedioxythiophene), protic ionic liquids, stability

## Abstract

Poly(3,4-ethylenedioxythiophene):poly(4-styrenesulfonate) (PEDOT:PSS)-based organic electrochemical transistors (OECTs) are widely utilized to construct highly sensitive biosensors. However, the PSS phase exhibits insulation, weak acidity, and aqueous instability. In this work, we fabricated PEDOT OECT by alternating current electrodeposition in protic ionic liquids. The steady-state characteristics were demonstrated to be stable in long-term tests. In detail, the maximum transconductance, the on/off current ratio, and the hysteresis were stable at 2.79 mS, 504, and 0.12 V, respectively. Though the transient behavior was also stable, the time constant could reach 218.6 ms. Thus, the trade-off between switching speed and stability needs to be considered in applications that require a rapid response.

## 1. Introduction

Because of their biocompatibility, intrinsic amplification capability, and low operation voltage, organic electrochemical transistors (OECT) are widely used in biosensing [1,2,3] and electrophysiology recordings [4]. As a p-type semiconductor, poly(3,4-ethylenedioxythiophene) (PEDOT) is by far the most used organic polymer to construct OECT [5]. The charge carriers of PEDOT are mobile holes, which can hop from one chain to another. Poly(styrene sulfonate) (PSS) is intrinsically hygroscopic and widely used as a dopant in the form of anion. On one hand, the dopant enables solution processing for PEDOT and provides channels for ions transport in the blend. On the other hand, the PSS phase, however, deteriorates the device stability in the presence of moisture and has weak acidity, which is harmful to surrounding materials such as flexible substrates. Therefore, new families of anions have been proposed. For instance, Inal and co-workers incorporated (trifluoromethylsulfonyl) sulfonylimide (TFSI) into polystyrene (PS) backbones [6]. The performance of PEDOT:PSTFSI-based OECTs appeared similar to that of state-of-the-art PEDOT:PSS devices. Moreover, some researchers tried to remove the PSS chains from polymeric matrixes. Ouyang’s group reported that a strong acid (H_2_SO_4_) socking treatment of PEDOT:PSS film improved OECTs stability [7]. An acid pre-doping method was also proposed recently. Adding of H_2_PtCl_6_ to a pristine PEDOT:PSS solution led to phase separation between PSS and PEDOT and resulted in the formation of a neutral phase, PSSH [8].

The most used method for OECTs fabrication combines photolithography and spin-coating [9]. Printing techniques, such as screen and inkjet printing, are also widely investigated [10]. We fabricated PEDOT:PSS OECTs by the alternating current (AC) electrodeposition method [11]. Using this method, a PEDOT:PSS film connecting the source and the drain was prepared on a non-conductive surface, and the OECT could be readily fabricated in situ without requiring the post-bonding process necessary for the conventional construction of microfluidic chips. However, the electropolymerization potential of 3,4-ethylenedioxythiophene (EDOT) monomers is similar to [12] or even exceeds [13] the electrochemical window of water. This would damage planar electrodes and yield unwanted reaction products. A ionic liquid (IL) is entirely composed of ions, exhibits the advantage of wide electrochemical windows, and hence, is widely used in electrodepositions. Till now, metals such as Ag [14] and Cu [15], semiconductors such as Si [16] and Ga [17], as well as alloys such as NiCo [18] and ZnMn [19] have been successfully electro-synthesized in ILs. Electropolymerization of organic polymers like PEDOT in IL without PSS doping has also been reported. For example, Serafín [20] synthesized PEDOT on carbon electrodes using the ionic liquid 1-butyl-3-methylimidazolium hexafluorophosphate in a potentiostat manner. A PEDOT film electro-polymerized by cyclic voltammetry in 1-ethyl-3-methylimidazolium bis(trifluoromethylsulfonyl)imide was used as the antifouling surface of biosensors by Luo’s group [21]. The influence of the solvent on the structure, morphology, and electrochemical properties of the polymer films was investigated by Wang and co-workers [22].

ILs are generally classified into two categories, protic ionic liquids (PILs) and aprotic ionic liquids (AILs). The research of electrodeposition in PILs is still in its infancy and is receiving increasing attention because of its advantages of low cost and low toxicity. Recently, the PIL hexylammonium(bis (trifluoromethylsulfonyl)imide) (HHexam(Tf_2_N)) was synthesized by our group [23,24]. The physicochemical properties such as density, viscosity, and electric conductivity of the PIL were investigated systematically. Here, we report for the first time the template-free AC electrodeposition (ACED) of PEDOT films in a PIL. The PEDOT films prepared on a non-conductive substrate were utilized as semiconductors to construct OECTs. Because no PSS was used in the semiconductor, the OECTs showed good water stability. Moreover, the PEDOT OECTs exhibited depletion-mode and p-type characteristics with good steady-state performance, which is promising for their application in high-sensitivity biosensors in the future.

## 2. Results and discussion

### 2.1. Chemical and Morphology

**Figure 1:**
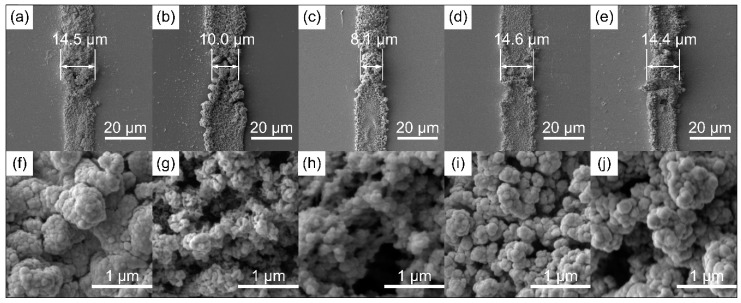
Scanning electron microscope (SEM) image of poly(3,4-ethylenedioxythiophene) (PEDOT) films electropolymerized in hexylammonium(bis (trifluoromethylsulfonyl)imide). The alternating current (AC) parameters used in (**a**,**f**) were 5.0 V_p-p_ and 50 Hz; in (**b**,**g**) 5.0 V_p-p_ and 75 Hz; in (**c**,**h**) 5.0 V_p-p_ and 100 Hz; in (**d**,**i**) 5.5 V_p-p_ and 50 Hz; in (**e**,**j**) 6.0 V_p-p_ and 50 Hz. A SEM image without electrodeposition is shown in Figure S1.

Figure 1 shows the scanning electron microscope (SEM) image of PEODT films ACED from the protic ionic liquid. As shown, the films consisted of aggregates of micro/nanoparticles, and their average width decreased with increasing AC frequency (Figure 1a–c). The mechanism of their formation could be elucidated considering the electrophoresis process, which was influenced by the electric field distribution. In detail, the ACED of PEDOT began at the initial oxidation of EDOT monomers [25,26,27]. Then, radical cations of oligomers formed and drifted in the IL. Because the electric field was uneven around the electrode tip and in the electrode gap [11], the electrophoretic velocity of the radical cations varied at different spatial positions. Besides, the electric double layers only undertook a part of the open-circuit voltage, and the un-screened fraction increased with the increasing frequency [28]. Thus, the radical cations were more inclined to distribute with respect to the electric-field profile, and the PEDOT films exhibited a propensity to polymerize in a finer form at a higher frequency.

Figure 1 also illustrates that PEDOT films formed on the whole surface of microelectrodes. In contrast, in aqueous solutions at high-frequency AC potentials, only the electrodes tips were coated [11]. At higher magnifications, the films exhibited a compact and globular morphology when the frequency was 50 Hz and the amplitude was 5 V_p-p_ (Figure 1f). As the frequency increased to 75 Hz, there was an increase in the porosity of the sample (Figure 1g). A spongy nature of the surface was observed when the frequency was 100 Hz (Figure 1h), which was consistent with the experimental results in Ref. [29]. This was further confirmed by the morphologies obtained under different AC amplitudes when the frequency was fixed (Figure 1f,i,j). As shown, the films became more poriferous when the amplitude was increased from 5.0 V to 6.0 V, while the average width did not change significantly.

**Figure 2:**
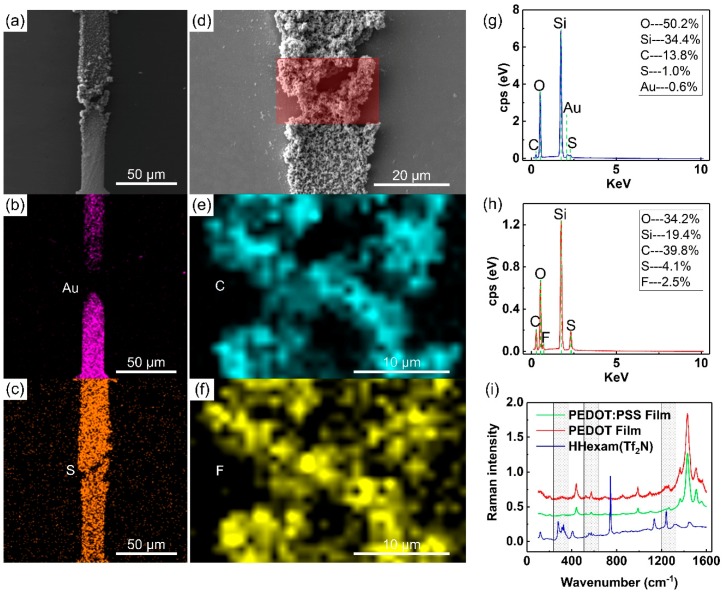
Analysis of the chemical composition based on the energy dispersive spectra (EDS) and Raman spectra. (**a**) SEM image of the film obtained by an AC voltage of 5.5 V_p-p_ and 50 Hz. (**b**) EDS mapping of the image in (**a**), the element was gold. (**c**) EDS mapping of the image in (**a**), the element was sulfur. (**d**) Magnification of the image in (**a**) and selected area for EDS investigations. (**e**) EDS mapping of the selected area in the image in (**d**), the element was carbon. (**f**) EDS mapping of the selected area in the image in (**d**), the element was fluorine. (**g**) EDS spectra of the image is (**a**). (**h**) EDS spectra of the selected area in the image in (**d**). (**i**) Raman spectra of HHexam(Tf_2_N), PEDOT film, and PEDOT:poly(styrene sulfonate (PSS) film. Raman bands of the PEDOT:PSS film (red curve) obtained previously and reprinted by permission from Springer Nature [11].

It is known that the operation of OECTs relies on concurrent electronic and ionic transport in the semiconductor layer. Thus, revealing the chemical composition of electrodeposited films may enrich our understanding of structure–property–function relationships [30]. Figure 2a–h show the energy dispersive spectrometer (EDS) mapping and spectra of PEDOT films. As shown in Figure 2b,c, the films formed onto the electrode surface, as well as into the electrode gap. In detail, the EDS mapping of gold defined the microelectrodes (Figure 2b). The EDS mapping of sulfur defined the film region because both PEDOT and the PIL molecules contained this element (Figure 2c). Besides, fluorine’s distribution evidenced the presence of PIL anions. When a large scan area was selected, the peak of fluorine was overwhelmed by oxygen and silicon contained in the substrate (Figure 2a,g). However, the atomic ratio of fluorine could be up to 2.5% when a smaller mapping area was selected (Figure 2d,h). As shown in Figure 2e,f, both fluorine and carbon were confined in the prepared films, but only [Tf_2_N]^−^ contained the element fluorine. Thus, we speculated that [Tf_2_N]^−^ was co-electrodeposited with the radical cations [EDOT]_n_^+^.

Then, Raman spectra were recorded to verify the assumption (Figure 2i). In the Figure 2i, the red curve indicates the vibrational bands of the PEDOT film ACED in the PIL. Compared with spectra of PEDOT:PSS films [11] ACED in aqueous solution (green curve), there was no significant changes in the peaks at 1430 cm^−1^, 1366 cm^−1^, and 989 cm^−1^, which were attributed to the symmetric C_α_=C_β_ stretching, C_β_–C_β_ stretching, and oxyethylene ring’s deformation, respectively. However, the PEDOT film exhibited a broader band at 1230–1270 cm^−1^. It could result from the effect of the peak at 1241 cm^−1^ of [Tf_2_N]^−^ anions (blue curve), which could be assigned to the symmetric CF_3_ stretching and deformation. Besides, the red curve exhibited a more intensive peak around 525 cm^−1^, and a new peak around 317 cm^−1^, which also could be due to the contribution of vibrational bands of the PIL. In addition, peaks attributed to the cations of the PIL (blue curve), such as those at 1440 cm^−1^ and 1330 cm^−1^, were not present in the PEDOT film spectrum (red curve). Thus, the above analysis indicated that [Tf_2_N]^−^ was co-electrodeposited in the PEDOT film in the experiment. The result is consistent with those of Luo’s work [31], where [Tf_2_N]^−^ was relatively small. As shown, the PEDOT bands overlapped with the PIL bands, such as that at 1131 cm^−1^ to that of ${\nu (\mathrm{SO}}_{2})$, that at 744 cm^−1^ to that of CF_3_, and that at 405 cm^−1^ to that of δ(SNS) in the red curve, which is consistent with the EDS results that only a small amount of fluorine was present in the PEDOT film.

### 2.2. Long-Term Performance

**Figure 3:**
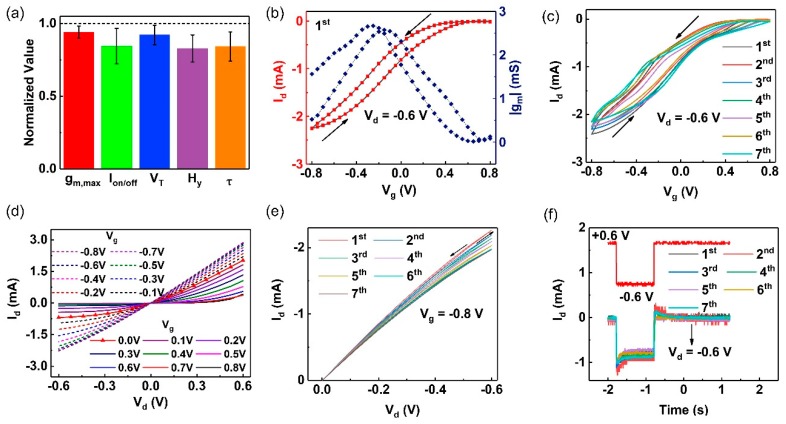
(**a**) sSability test during 150 h. Steady- and transient-state tests were conducted every 24 h. The error bar indicates the SD (n = 7). (**b**) Transfer characteristic curves (red line) and the transconductance curves (blue line) for V_d_ = −0.6 V. (**c**) Hysteretic transfer curves for V_d_ = −0.6 V. (**d**) The output characteristic curves for V_g_ increased from −0.8 V to 0.8 V. (**e**) Hysteretic output curves for V_g_ = −0.8 V. (**f**) Transient response under a square-wave V_g_ from −0.6 V to 0.6 V.

The procedures to build the OECT are illustrated in the “Material and Method” section. In brief, we prepared microelectrode chips by the Micro-Electro-Mechanical System (MEMS) process. PEDOT films were electrodeposited in situ on the silicon dioxide to connect the source and the drain electrodes. Then, the OECT was constructed using microelectrodes, PEDOT films, a sodium chloride solution, and an Ag/AgCl electrode. For stability characterization, the OECT was immersed in a 100 mM NaCl solution. Steady-state and transient-state experiments were performed every 24 h. Figure 3a illustrates that the OECT, which was constructed using PEDOT films prepared by an AC voltage of 50 Hz and 5.5 V_p-p_, exhibited good stability against moisture up to 150 h.

The transfer characteristic curves were obtained by scanning gate bias (V_g_) from −0.8 V to 0.8 V when the drain potential (V_d_) was fixed at −0.6 V (Figure 3b). As shown, the OECT operated in depletion mode. The maximum transconductance (g_m_) approaching 2.57 mS was obtained when V_g_ was −0.15 V in the forward scan, while it reached 2.64 mS at V_g_ = −0.25 V in the backward scan. The forward and backward threshold voltage (V_T_) could be obtained by fitting the linear region of I_d_^1/2^–V_g_ curves (Figure S2), defined as 0.65 and 0.53 V, respectively. Generally, OECTs exhibit large hysteresis (H_y_) because the doping and dedoping processes are three-dimensional. For the quantitative expression, H_y_ was defined as the difference between the forward and the backward V_T_. Figure 3a,c illustrate that the hysteretic transfer curves exhibited good repeatability throughout the week. H_y_ was estimated at around 0.12 V, as shown in Table S1 in the Supplementary Materials.

The output curves were obtained by scanning V_d_ from +0.6 V to −0.6 V when the V_g_ was increased from −0.8 V to 0.8 V with a step of 0.1 V (Figure 3d). The depletion of holes under positive V_g_ and negative V_d_ could be clearly observed. Figure 3e illustrates the stable output characteristic and the negligible hysteresis throughout the long-term tests. As shown, the deviation of currents in the source-drain loop (I_d_) only increased when a larger V_d_ was used. Besides, a stable on/off current ratio up to 504 was obtained (Table S1), which would help to reduce the detection limit of OECT-based biosensors. Figure 3f illustrates the transient response curves. As shown, the switching-on/switching-off time was up to 205.1 and 218.6 ms, respectively. The response speed was slower than that of devices of the same size fabricated by spin-coating [32] or printing [33]. It was also slower than that of the PEDOT:PSS OECT, ACED in aqueous solution [11].

In sum, the amplification capability of PEDOT OECTs was comparable to that of PEDPT:PSS/graphene quantum dots we reported previously [11]. This may be due to the porosity of the electrodeposited films, which could be beneficial to the transportation of liquid molecules. This may also be because removing the insulating PSS phase increased the conductivity of the semiconductor layers. The removal of the PSS phase was shown to be beneficial to device stability because the delamination phenomenon could be eliminated. However, the PSS phase is hydrophilic and thus could facilitate the transportation of solvated molecules. Thus, PEDOT OECTs exhibited a lower transient speed, and high-frequency applications such as electrocardiogram recordings may be limited.

## 3. Materials and Methods

### 3.1. Chemicals

EDOT was purchased from Alfa Aesar (Alfa Aesar Corp., Ward Hill, MA, USA). The PIL was synthesized according to our previous work [23]. In detail, hexylamine (Hexam) and bis(trifluoromethylsulfonyl) imide (HTf_2_N) were mixed in diethyl ether for 4 h. Then, the PIL HHexam(Tf_2_N) was obtained by complete evaporation of diethyl ether as a colorless transparent liquid at room temperature. HTf_2_N (>99%, CAS 82113-65-3) and hexam (>99%, CAS 111-26-2) were supplied by Nanjing Duolun Chem. Co. Ltd (Nanjing, China). Diethyl ether (>99.5%, CAS 60-29-7) was supplied by Beijing Chem (Beijing, China). Work and was distilled after soaking in molecular sieves overnight. The density of the PIL was 1.4124 g·cm^−3^, its viscosity was 159 mPa·s, and its conductivity was 530 μS·cm^−1^ measured at 308.15 K.

### 3.2. ACDE on Microelectrodes Chip

An Si wafer with a 500 nm thermally grown SiO_2_ film served as the substrate. A 20 nm-thick Al film was deposited as the cohesive layer for a 500 nm-thick Au layer. The chip was lifted off in acetone, and microelectrodes (source, drain) formed. Then, the microelectrodes chip was fixed onto a printed circuit board by adhesive tape. Gold wires of 200 μm in diameter were used to connect the microelectrode pads with the PCB board for external electrical connections. The ACED process was similar to that reported in our previous work [11]. Then, 10 mM EDOT was dissolved in 0.5 mL HHexam(Tf_2_N). The mixture was put into an ultrasonic water bath for 1 h, and 2 μL of the mixture was transferred onto the microelectrodes chip by a microsyringe. The arbitrary function generator AFG2021-SC (Tektronix Inc., Beaverton, OR, USA) was used to provide AC voltage between the source and the drain. Once the film was formed, the AC power was switched off manually.

### 3.3. Morphology, Structure, and OECTs Characterization

SEM characterization was carried out by TASCAN, LYRA3 (Brno, Czech Republic) at an acceleration voltage of 10 kV, and the element composition was obtained via the attached EDS. The vibration spectra were obtained by using a laser Raman spectrophotometer (LabRAM HR Evolution, HORIBA Jobin Yvon, Paris, France) with a green laser. The selected wavelength was 532 nm, and the laser power intensity was 13 mW·(cm^2^)^−1^. All measurements were conducted within 30 s accumulating one time and utilizing 5% of the laser power. To characterize the electrical properties, the surfaces of the source and the drain not covered by electrodeposits were insulated by ultraviolet-curable adhesive (NOA74, Norland Adhesives, Hackensack, NJ, USA) manually. A 100 mM NaCl solution was utilized as the gate electrolyte medium. An Ag/AgCl electrode with a negligible potential drop was immersed into the NaCl solution and served as the top-gate electrode. The electrode was at a distance of 200 μm on top of the microelectrodes chip. The steady-state characteristics of OECTs, such as transconductance and threshold voltage, were measured using a 2636B source meter (Keithley Instruments, Cleveland, OH, USA). The transient-state characteristic of OECT was measured by the current preamplifier SR570 (Stanford Research Systems, Sunnyvale, CA, USA), sampling with the data acquisition panel 6343 (National Instruments, Austin, TX, USA). The current in the source-drain loop was measured on the drain side, whether in transient-state or steady-state investigations.

## Supplementary Materials

SEM image of electrodes without electrodeposition; long-term hysteretic transfer curves; data obtained from the long-term tests.
