# Supplementary material for: AC Electrodeposition of PEDOT Films in Protic Ionic Liquids for Long-Term Stable Organic Electrochemical Transistors
Source: Molecules. 2019 Nov 14;24(22):4105. doi: 10.3390/molecules24224105 (PMC6891491; doi:10.3390/molecules24224105)
Supplement: Supplementary file 1 [file molecules-24-04105-s001.pdf]

AC Electrodeposition of PEDOT Films in Protic Ionic Liquids for Long-Term Stable Organic Electrochemical Transistors

Jianlong Ji [1,*], Xiaoxian Zhu [1], Dan Han [1], Mangmang Li [1], Qiang Zhang [1], Yang Shu [2], Zhengdong Cheng [3], Wendong Zhang [1], Er Hua [4,*] and Shengbo Sang [1,*]

1 College of Information and Computer, Taiyuan University of Technology, Taiyuan 030024, China; zhuxiaoxian622@163.com (X.Z.); hd06520101@163.com (D.H.); 18634391812@163.com (M.L.); zhangqiang01@tyut.edu.cn (Q.Z.); wdzhang@tyut.edu.cn (W.Z.)
2 Department of Chemistry, Colleges of Sciences, Northeastern University, Shenyang 110819, China; shuyang@mail.neu.edu.cn
3 Artie McFerrin Department of Chemical Engineering, Texas A&M University, College Station, TX 77843-3122, USA; zcheng@tamu.edu
4 Chemical Science and Engineering College, North Minzu University, Ningxia 750021, China
* Correspondence: jijianlong@tyut.edu.cn (J.J.); huaer0101@hotmail.com (E.H.); sunboa-sang@tyut.edu.cn (S.S.)

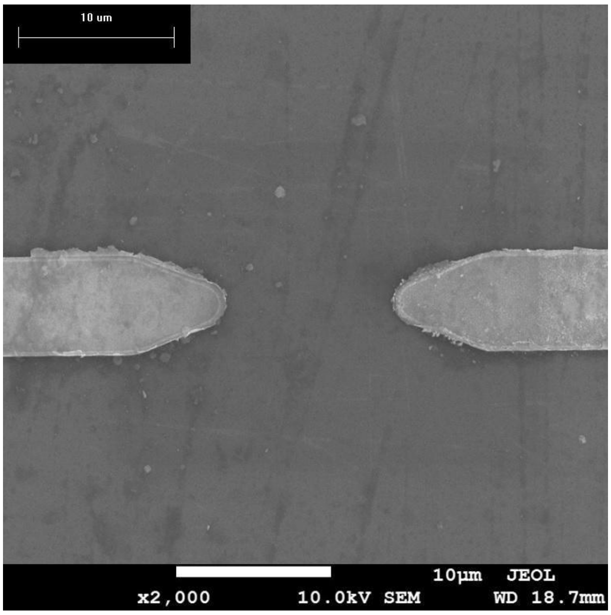

Figure S1. SEM image of the electrodes without electrodeposition

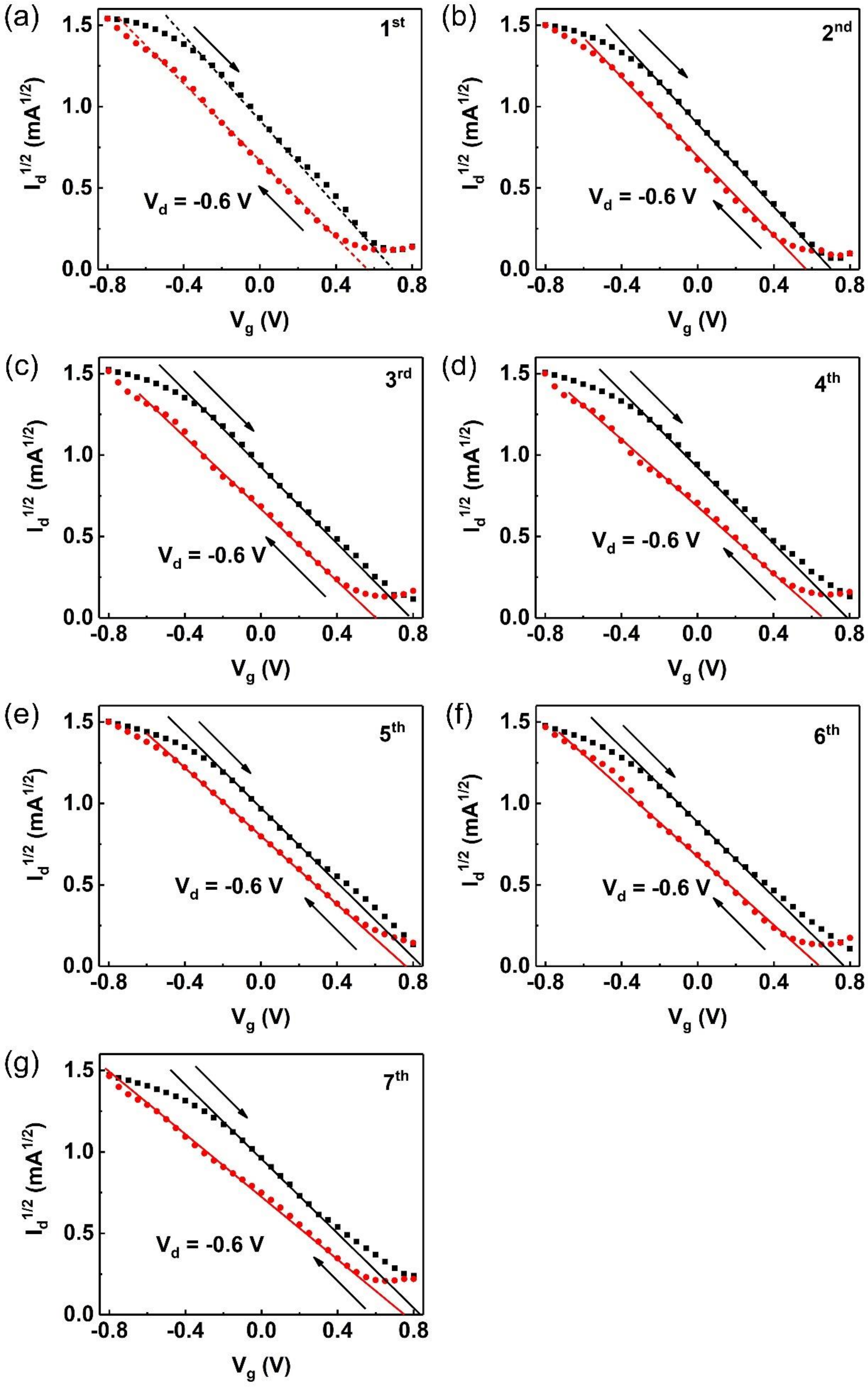

Figure S2. Hysteretic transfer curves for V_d= -0.6 V within one week.

Table 1. Steady- and transient-state characteristics of the organic electrochemical transistor (OECT) in long-term investigations.

	**1st**	**2nd**	**3rd**	**4th**	**5th**	**6th**	**7th**	**Mean ± SD**
**$\|g_{m,max}\|$ (mS)**	2.57	2.76	2.80	2.76	2.81	2.88	2.97	2.79 ± 0.12
**I_{on}/I_{off}**	575.0	497.9	477.8	544.8	395.5	442.1	597.3	504.3 ± 72.7
**V_T (V)**	0.67	0.70	0.76	0.78	0.82	0.75	0.81	0.75 ± 0.05
**H_y (V)**	0.12	0.13	0.15	0.12	0.11	0.13	0.11	0.12 ± 0.01
**τ_{on} (ms)**	214	232	228	186	202	182	192	205.1 ± 20.0
**τ_{off} (ms)**	226	205	232	219	210	230	208	218.6 ± 11.1
